# Interacting With Competence: A Validation Study of the Self-Efficacy in Intercultural Communication Scale-Short Form

**DOI:** 10.3389/fpsyg.2020.02086

**Published:** 2020-09-04

**Authors:** Russell S. Kabir, Aaron C. Sponseller

**Affiliations:** ^1^Graduate School of Humanities and Social Sciences, Hiroshima University, Hiroshima, Japan; ^2^Department of International and English Interdisciplinary Studies, Osaka Jogakuin College, Osaka, Japan

**Keywords:** self-efficacy, intercultural communication, self-regulated learning, L2 motivation, sojourner self-efficacy in communication

## Abstract

Self-efficacy encompasses the professional and personal language goals of learners as their progress depends upon a strong motivation to put practical language skills to use when the real world requires it. Intercultural communication and effectiveness are of interest to the professional and personal language goals of learners as their progress depends upon a strong motivation to put practical language skills to use when the real world requires it. Studying or working abroad and engaging in intercultural training are two such contexts that bind research in learner characteristics between applied linguistics and positive psychology as they provide a substrate of concrete interactions, transformative experiences characterized by opportunities for changes in self-concept, negotiations with values and authenticity, and forms of interpersonal development underwritten by intercultural communication as an ability. A tool to capture this domain-specific intercultural communication was previously developed with sojourner educational professionals for use among English speaking populations. However, the original study lacked confirmatory analyses of internal and external validity that would clarify model identification and applicability for research that deals with intercultural communication competence across populations with diverse sample characteristics. A total of 876 teachers (*M* age = 37.48, *SD* = 10.81) and 266 university students (*M* age = 19.48, *SD* = 0.74) in Japan responded to items from the SEIC instrument. Acceptable model fit was supported for the eight-item short form. Metric invariance was observed for individuals from a sample of sojourning English language teachers similar to the original validation and a nationwide survey of Japanese teachers of English, offering indications of cross-cultural validity. Degrees of equivalence were also found for the Japanese items as extending fitness for use to students from two universities in Japan. Concurrent validity was supported for SEIC measured by the scale with intercultural effectiveness competencies and speaking and listening self-efficacy constructs used in classroom contexts. Together, this study offers a tool of valid indicators for researchers and practitioners who aim to observe self-efficacy in positive education, intercultural training, or international programs that intersect with language learning and intercultural communication.

## Introduction

Communicative competence is a penultimate goal for language learners. Numerous theories have been posited for its instructional and developmental processes, but tools for applications, such as simulations and learning experiences designed to augment communicative competence mediated by language, require added considerations for domain specificity. While some instruments have been established, they typically aim to capture micro processes from classroom use cases or higher-order macro beliefs about sociocultural adjustment. Thus, many constructs lack the needed focus for a mid-level construct that considers situated interactions with interlocutors marked with sociocultural proficiency. Gains in self-efficacy in communication and language can be made from experiential learning, which serves as a connective tissue for work in education with positive outcomes. A nomological net for self-efficacy in intercultural communication (SEIC) was carefully identified with convergent and discriminant analytic techniques by Peterson et al. (2011), which provides a framework rooted in social cognitive theory and a mid-level construct recognized as necessary for practitioners (Lake, 2016). Their study showed potential applications for SEIC in a sample of professionals with overseas sojourning and teaching experience. However, the original scale development attempt used a relatively modest sample size (*N* = 213) of former sojourning teachers and ended with preliminary checks for internal validity, likely contributing to some hesitancy to select or adopt the instrument in related assessment research (Goldstein, 2015). This report offers numerous positions in favor of the approach and instrument taken by Peterson et al. (2011) with further theoretical support due to the usefulness of an SEIC construct in applied settings and addresses questions of validity to evaluate its potential as a tool for evaluators and educators.

### Overall Communicative Competence and Intercultural Learning

Long been the target of classroom instruction, overall communicative competence is theorized as the chief goal for learners in terms of function, discourse, register, non-verbal human communication and linguistic negotiation (Yorio, 1976; Paulston, 1992; Brown, 2000). Constructivist theories emphasize the role and dynamism of interactions (Long, 1996) as a matter of language transactions and extend such notions to include key elements of awareness, autonomy, and authenticity in theories of practice for pedagogical design (Van Lier, 2014). Speakers and listeners chisel and achieve competency through interactions and their feedback. Furthermore, cultural knowledge and awareness enhance the resolution of the available and practical language lexicon and contextualizes it with ecologically valid situational knowledge (Sample, 2013; Rebstock, 2017; Yoshida et al., 2018). In this way, skilled flexibility, variability, and familiarity with language features such as style and register are needed for communicating with attendant illocutionary force (Brown, 2000).

Intercultural learning allows for individuals to become competent with these features by being aware of multiple perspectives and facilitating long-lasting personal growth. Simulation activities such as “Rocket” (Hirshorn, 2009; Kirchhoff and Yabuta, 2017) or exercises with active learning and discussion of critical incidents are rich in implementation fidelity, structured instruction, and the ability to grant learners with unique opportunities to obtain intercultural awareness (Wilson, 2017; Yoshida et al., 2018). Among developmental events for emerging adults, the study abroad or sojourn experience is considered prototypically “life-changing” as it provides genuine opportunities for individuals to negotiate meaning through engagement with their own mind and body against the backdrop of a given surrounding culture, society, and its members. Insights from the psychology of happiness through travel support the notion that international sojourns affect personal growth through multicultural encounters in a transformative fashion (Couper, 2001; Filep, 2009), and transformative experiences are associated with esthetic appraisals and the virtue of transcendence in applied positive psychology (Keltner and Haidt, 2003; Lomas et al., 2014). In reference to the conceptual map of applied positive psychology by Lomas et al. (2014), the sojourn specifically offers paths to subjective well-being through encounters with material substrates in the built environment (e.g., architectural marvels) as physical objects with collectively shared meaning, as well as new interpersonal encounters mediated *via* communication (e.g., everyday transactions with others from immersion into the host culture) as relational experiences. Other implicit judgments and interpersonal interactions are known to provide instrumental outcomes and exposure to norm representations from feedback after immersive experiences (Morris et al., 2015). In this way, sojourns provide those with values or strengths such as open-mindedness, curiosity, love of learning, or others (Deardorff, 2006) the opportunity to negotiate expectancies and arbitrate their signature status and perhaps even develop their sense of authentic personality (Wood et al., 2008) through expansions in cultural understanding from interactive behavior. In this manner, expanding cultural understanding through awareness, knowledge, emotions, and skills is a strong precursor to the sojourner efficiency and experiential learning that stands as an exemplary feature of intercultural training (Rebstock, 2017; Yoshida et al., 2018).

### Intercultural Communicative Competence With Interlocutor Interactions as Integral Domain

The strides in self-concept made from exposure to new environments and perspectives through interactive behaviors come from concrete experiences under intercultural circumstances, and these inform beliefs about the competence that an individual possesses to navigate communicative encounters with aplomb. Researchers have dubbed this ability *intercultural communication competence* (ICC). According to the definition by Fantini and Tirmizi (2006), ICC represents “the complex of abilities needed to perform effectively and appropriately when interacting with others who are linguistically or culturally different from oneself” (p. 12; Godwin-Jones, 2013). Properties of ICC were carefully qualified using the Delphi technique (Deardorff, 2006) and categorized into 24 agreed-upon components. These were summarized into a conceptual pyramid that builds upward from a substrate of diffuse attitudes and coalesces into degrees of acquired knowledge of culture and language skills, leading into internalized adaptations of new communicative styles, and ultimately peaks with an actualized ability to demonstrate effective and appropriate behavior *vis-à-vis* communication within and between cultures. The components have been operationalized for assessing student outcomes (Deardorff, 2011) and paved the way for efforts to capture and track changes in intercultural effectiveness (Nguyen, 2017) from an inventory of global competencies (Mendenhall and Osland, 2002). A process model of ICC utilizing the same components as the pyramidal model was also conceptualized to illustrate the objects of incremental and cyclical ICC development. Notably, researchers have called for more contextually diverse explorations into ICC due to the general locus of input from Western institutions (Deardorff, 2015; Deardorff and Arasaratnam-Smith, 2017) and expressed concern that “current models of intercultural competence do not sufficiently address the role of language competence in intercultural competence” (Deardorff and Arasaratnam-Smith, 2017, p. 299). In contrast, the SEIC covers both aspects of these competencies and incorporates ICC content in Deardorff (2006) that encompasses sociolinguistic awareness. Additionally, the instrument project by Peterson et al. (2011) was explicitly cited as one of the few examples of communicative studies where sojourners underwent supervision overseas in a non-Western host culture (Simmons, 2014).

Studies have clarified that desired internal and external outcomes exist for gains in sociocultural understanding such that “effectiveness” in communication is informed by culturally attuned knowledge and manifested behaviorally by achieving goals (i.e., the Pyramid Model of Intercultural Competence; Deardorff, 2006) but also by demonstrating the zenith of communicative behavior (Yashima et al., 2016). Development of the SEIC was originally based on the suppositions of intercultural transformation theory (IFT) to which communicative encounters are thought to be evaluated as beliefs about competence while under the duress of intercultural interactions. IFT draws strong parallels from the cyclical process model of stress-adaptation-growth by Kim (2001) and advances the notion that intercultural interactions that do not conform to sojourner expectations become a wellspring for intercultural growth. Non-conforming interactions lead to disequilibration (e.g., stress), to which the sojourner responds to new and alternative cultural norms and is faced with the choice to integrate them into their intercultural repertoire (e.g., growth) as a matter of adaptive behavior. As the iterations of non-conforming intercultural interactions mount over time, greater degrees of adaptation hypothetically build within the sojourner. In a more countable fashion, each iteration of non-conforming interaction is essentially an appraised stressor that Experiential Learning Theory (ELT) by Kolb (1984) would define as a *concrete experience* that invites sojourners to reflect, reconceptualize, and experiment with their understanding the next time that they find themselves in a similar situation.

Nguyen (2015) recognized and illustrated that the longitudinal stress-adaptation-growth cycle and more cross-sectional ELT cycle contain substantial and intuitive theoretical overlap vis-à-vis concrete experiences. The primary commonality across the aforementioned models (intercultural competence, ELT, stress-adaptation-growth) for beliefs in linguistic capability among sojourners is the notion that episodes of interpersonal engagement are critical to initiate possible growth within individuals. ELT, as a cyclical theory, requires such experiences to begin a cycle and stipulates that students take ownership of the learning experience. In study abroad experiences, especially, building diverse learning relationships with individuals while abroad is emphasized as a means to “promote growth and movement through the learning spiral” (Passarelli and Kolb, 2012, p. 156). Analogously, the everyday nature of human concerns is argued as a key advantage for leveraging techniques in *positive education*, as the topics of positive psychology encompass the emotional lives of students whose personal experiences and positive emotions can be called upon for reflection in expressive ways (Biswas-Diener and Patterson, 2014). In this way, positive education is similarly experiential and reliant on learner emotions and beliefs as a basis for awareness and self-regulation. Managing learner emotions was directly targeted in work on emotional regulation strategies in language learning with vignettes (Gkonou and Oxford, 2016) and even investigated for implementation fidelity with techniques in Cognitive Behavioral Therapy (CBT) (Curry et al., 2020). Moving beyond the classroom, positive education applications to intercultural communication competence would be salient for the transformative experiences that are thought to accompany sojourning for work or study, calling upon methods in reflective and experiential learning (Kolb et al., 2001).

Psychological adjustment is well-understood by stress and coping frameworks (Li and Gasser, 2005), and IFT appears adequate in its approach to capturing elements of the stress-adaptation-growth cycle. However, we also advocate for contemporary research that more explicitly integrates the notion of a substrate of interactions with Kolb’s experiential learning cycle (Passarelli and Kolb, 2012) and an addendum that engaging in interactions requires a willingness to communicate (WTC). Proponents of WTC contend that linguistic variables (communicative competence, L2 self-confidence, and state-communicative self-competence) and social and situational variables (intergroup climate, social situation, intergroup attitudes, intergroup and interpersonal motivation, and the desire to communicate with a specific person) form the interactive substrate for these outcomes (MacIntyre et al., 1998). In the Japanese context, self-perceived communicative competence was the most robust correlate with WTC in English (*r* = 0.56) among Japanese undergraduates (Yashima, 2002) and was the only statistically significant correlate (*r* = 0.46) among Japanese high school students preparing to study abroad for a year (Yashima et al., 2004). Spoken WTC was found to be significantly influenced by the perceived ability to speak in English among a sample of 1,789 Japanese university students, and differences were discovered to depend on the type of interlocutor (Japanese student, Japanese teacher, non-Japanese student, non-Japanese teacher) encountered (Weaver, 2010). Each of these studies offers support for the WTC model, and lends credence to the notion that state-like components of L2 self-efficacy are integral in the choice of whether to engage in an interaction. Additionally, research on language learning holds sufficient promise as a concrete stressor for appraisal and coping in the specified domain but requires further rigor in approaches to measurement of constructs with cross-cutting and positive trait-related implications (Lazarus, 2003; Dewaele et al., 2019).

### Interacting With Competence: Learning Through Self-Efficacy and Intercultural Communication

Evaluating language and intercultural competence requires a belief in one’s capability to perform effective communication. Peterson et al. (2011) recognized that one of the most appropriate and available areas of focus for communicative competence that binds the relevant disciplines and research programs was through examinations of *self-efficacy*, a core construct that weaves between cross-cultural research in educational settings. Situated along the framework for the social cognitive theory of learning provided by Albert Bandura in the 1970s, self-efficacy encompasses a wide array of psychosocial components that incrementally contribute to levels of attainment toward goals. The first relates to modeling and obtaining experiences of mastery through direct engagement with tasks or their vicarious observation. The second involves the influence of positive or negative feedback of people in interpersonal interactions regarding the skill at hand, especially such that the communication emphasizes social persuasion. The last major component relates to the process of evaluating and regulating markers of internal or physiological states from the body while performing the skill or behavior (Bandura, 1997). Mak and Tran (2001) assigned direct applications of self-efficacy to language and intercultural competence according to the criteria put forth by Bandura (Li and Gasser, 2005), such that domain-specific self-efficacy for language learners involves engaging actively in controlled cross-cultural interactions of social significance, observing peers perform in these interactions, seeking constructive feedback, and overcoming emotional arousal to enhance performance. Self-efficacy has also been tagged as a factor influencing sojourner and intercultural adjustment (Hechanova-Alampay et al., 2002) for both domestic and international sojourners (Goldstein, 2015). In a meta-analysis reporting a moderate effect size, cross-cultural self-efficacy change from intercultural adjustment was observed longitudinally and opposed measures of anxiety (Wilson et al., 2013), indicating its crosscutting role as a situational factor in these applications.

Numerous theories of motivation in language learning have adopted components relevant to self-efficacy—from goal setting (Lee and Bong, 2019), self-regulation (Kim et al., 2015), notions of personal investment (King et al., 2019), and value expectancy (Mori, 2002; Loh, 2019) to the four skills in languages such as English (Wang et al., 2013). Beliefs about language aptitude have been tied to self-efficacy in communication (Yang, 1999), and a self-efficacy model of interpersonal communication competence was developed by Rubin et al. (1993), whose study provided concurrent relationships between self-efficacy and satisfying communication such that levels of self-efficacy affected ratings of interpersonal communication competence as a belief in one’s skill. In other areas of applied linguistics, the WTC model has been studied extensively and in conjunction with measures of L2 self-confidence, which scholars like Dörnyei and Kormos (2000) have argued is a form of task-related or state-specific self-efficacy, especially in a study that showed L2 self-efficacy and relationship to the interlocutor positively correlated with the frequency of turn initiation in L2 discussions (Dörnyei and Kormos, 2000). However, L2 self-confidence and self-efficacy contain differences in the scope of content validity and the desired use by practitioners. The need for capturing an intermediate construct level domain for L2 self-efficacy was identified as a potential crossover construct by Dewaele (2012) and underscored in the work by Lake (2016). In terms of practical need for instructors and researchers of simulations, formal frameworks with common constructs have been similarly dubbed lacking and necessary for endpoint evaluation and evidence-based use of the exercises (Wiggins, 2012).

Training simulations extend to professional settings and serve as a throughline for ability benchmarking and intercultural skill acquisition. Professional self-efficacy is a key factor in theories of burnout (Maslach et al., 2001) that has received attention in research on teacher emotions (King et al., 2020). SEIC could be a target outcome for classroom contexts but also professional settings. The original sample population from Peterson et al. (2011) was composed of alumni participants of the JET Program. Thus, the SEIC study explored by Peterson et al. (2011) harnesses a strength in application in the form of its original focus on overseas professionals in educational settings. Continuing to secure a population with this upper bound might allow for observations of latent changes along the longitudinal arc of college student outcomes into ICC for educational professionals. For those earlier in training such as students obtaining work experience, service learning is a growing area where linguistic competence is meaningfully applied (Rebstock, 2017) and could be observed as a factor sensitive to longitudinal change. In fact, such implications for professional self-efficacy were a point of reference in a study of relevant attitudes by Harrison (2006), who acknowledged relationships between cultural connectivity and professional development, and in another study by Goldstein (2015) who reported shortcomings in variable selections from an implemented reliance of a general rather than domain-specific self-efficacy scale, specifically citing Peterson et al. (2011) as a logical next step for investigation. This is in line with the fact that, while formulations of the theory of self-efficacy have been pursued as a general capacity, task-related self-efficacy in applied psychology contexts is especially endorsed (Carr, 2013) and has been key for aligning with Bandura’s prescription that self-efficacy beliefs are best delineated under specific domains (Bandura, 2006). Overall, self-efficacy is an integral factor across these studies and research programs, and applications to intercultural communication further specify the domain. In this sense, SEIC is a versatile construct that spans the relevant disciplines and the expectations of learner gains. SEIC could conceivably find application inside formal classroom settings through formal instruction plans or outside of them in the form of real-life encounters (McEown and Oga-Baldwin, 2019) and even to professional development settings.

### The Present Study

In light of this reading of the literature, it is suggested that SEIC is a valuable domain and credible source of application to both professional and educational settings. However, the tool developed to evaluate SEIC only went through initial stages of validation with a limited sample size. The preliminary validation steps used principal axis factoring and Cronbach alpha-based reliability estimate comparisons to extract a measurement model of the items (Peterson et al., 2011) in a relatively modest sample (*N* = 213). The authors made efforts to establish an exploratory factor analysis-based structure, to which an eight-item short form of the instrument was established but noted that future studies remained necessary for confirming the factor structure and its generalizability.

Here, we apply the eight-item version proposed by Peterson et al. (2011) from data of teachers who sojourned to currently sojourning and host culture teachers and the 34-item original item version in a first-time application for undergraduate students. Our study is therefore motivated to address these areas of validation by (1) securing careful confirmatory analysis of the eight-item short form in a study design that shares sample characteristics of sojourning language teachers and extends beyond a single-language instrument through adaptation to another sample of Japanese teachers; and (2) attempting to replicate good fit for psychometric properties and nomological networks in samples of undergraduate students from two universities in Japan that would serve as targeted populations for the use of the instrument. These analyses are framed to posit that the construct can be attenuated in use cases of students motivated to benefit from sojourns but not necessarily exclusive to them as SEIC could extend to outcomes of intercultural training (e.g., simulations), calling for a localized form of the instrument that captures SEIC as a learner belief of interest to research and practice in applied positive psychology and linguistics.

## Materials and Methods

### Procedures and Study Participants

This study used cross-sectional survey designs to provide snapshots of relevant constructs for examining validity among a sample of native English language teachers sharing the characteristics of the original sample validation as well as native speakers of Japanese language who received and responded to Japanese adaptations of the items.

#### Sample of Teachers for Language Adaptation and Structural Validity

Paper-and-pencil questionnaires containing the short-form items proposed by Peterson et al. (2011) were distributed by postal mail to assistant language teachers (ALTs) and Japanese Teachers of English (JTEs) throughout Japan. A total of 876 teachers (*M* age = 37.48, *SD* = 10.81) responded to the survey section that included the eight-item short form instrument. Data from this sample were used only to examine questions of internal validity, to which listwise deletion for completed responses left 264 participating ALTs and 597 participating JTEs for analysis (*N* = 861). Sample details are depicted in Table 1.1.

**TABLE 1.1:** Demographic and descriptive information for the study variables for study participants from mail-in questionnaire data of the teachers.

Study variable	Sojourning Assistant Language Teachers (ALTs)	Japanese Teachers of English (JTEs)
Gender *(N,% Female*)	261* (48.66%)	590* (60%)
Female	127	354
Male	134	258
Age (*M*, *SD*)	28.7 (6.4)	41.3 (10.1)
Education (Highest Level Completed; *N*,%)		
Bachelor’s	225 (86.21%)	451 (76.44%)
Master’s	33 (12.64%)	107 (18.14%)
Doctorate	2 (0.77%)	6 (1.02%)
Years of experience (*M*, *SD*)		
Teaching	3.77 (3.67)	16.65 (10.21)
Team teaching	3.06 (2.97)	13.22 (9.09)
Currently team teaching (%)	96.55%	90.68%
Grade level teaching (*N*,%)		
Junior high school	118 (45.21%)	258 (43.73%)
High school	143 (54.79%)	332 (56.27%)
Self-Efficacy in Intercultural		
Communication (*M*, *SD*)	3.69 (1.20)	3.54 (0.82)

*Demographic data were missing or incomplete for three ALTs and seven JTEs, respectively. Item-level data of the fully completed SEIC items were retained for the analysis of structural validity. Average Self-Efficacy in Intercultural Communication (SEIC) scores were calculated for the eight-item version that was distributed to the samples of teachers.

Demographic data were provided by 261 ALTs (48.66% female; *n* = 127). The mean age of ALTs was 28.7 years, and they had been teaching English for an average of 3.77 years and team teaching (presumably in Japan) for 3.06 years. At the time of completing the survey, 96.55% said they were currently engaged in team teaching of English classes. Regarding teaching context, 45.21% (*n* = 118) taught at junior high schools, and 54.79% (*n* = 143) taught at high schools, respectively. Finally, concerning the highest formal level of education completed, 86.21% (*n* = 225) had completed a bachelor’s degree, 12.64% (*n* = 33) had completed a master’s degree, and 0.77% (*n* = 2) had completed a doctorate. The reliability coefficients for the SEIC among ALTs were α = 0.94, ω = 0.94.

Demographic data were provided by 590 JTEs (60% female; *n* = 354). The mean age of JTEs was 41.3 years, and they had been teaching English for an average of 16.65 years and team teaching for 13.22 years. At the time of completing the survey, 90.68% said they were currently engaged in team teaching of English classes. Regarding teaching context, 43.73% (*n* = 258) taught at junior high schools, and 56.27% (*n* = 332) taught at high schools, respectively. Finally, concerning the highest formal level of education completed, 76.44% (*n* = 451) had completed a bachelor’s degree, 18.14% (*n* = 107) had completed a master’s degree, and 1.02% (*n* = 6) had completed a doctorate. The reliability coefficients for the SEIC among JTEs were α = 0.90, ω = 0.90.

#### Sample of University Students as Target for Instrument Use

To investigate and validate relationships in a sample population targeted for use of the instrument, 266 students (*M* age = 19.48, *SD* = 0.74) from two different universities in Japan responded to questionnaires. Data were distributed and collected *via* an online survey research platform as a part of a pre–post study design akin to Nguyen (2017) to observe possible changes in intercultural effectiveness upon short-term study abroad program participation (duration: 2 weeks). Data from these samples were used to evaluate internal and external validity. All participants included gave their informed consent to participate and allowed the use of their data for analysis.

### Analytical Plan

Confirmatory factor analysis (CFA) is an approach to providing measurement models that systematically examine the structural validity of latent constructs (Brown, 2014). Continuing the next step in the validation process as acknowledged in the concluding sections of Peterson et al. (2011), CFA-based cross-validation was chosen to examine the generalizability of the factor structure of the short form of the SEIC.

Measurement invariance (MI) is an analytical approach that tests the degree of equivalence in relationships between responses to items and their associated latent constructs across groups (Pendergast et al., 2017). The three major steps of MI include checks for configural invariance, or whether the items are measuring the same factors across groups, metric invariance, or the degree that magnitudes of item–construct relationships are equivalent across groups, and scalar invariance, or whether the loadings and “threshold” intercepts are equivalent between groups (van de Schoot et al., 2012; Pendergast et al., 2017) for direct comparisons. MI was tested to determine degrees of internal and cross-cultural validity for the eight-item short-form instrument across groups.

Correlational analysis is used to understand convergent and divergent forms of external validity from the magnitude and direction of relevant latent constructs (DeVellis, 2016). Relationships between SEIC, language proficiency scores, and intercultural effectiveness at pretest as a cross-section were compared to further establish the content domain of the construct measured by the SEIC instrument. Coefficients above or between 0.2 and 0.4 were evaluated against theoretical assumptions and those especially exceeding 0.3 were deemed sufficient as intercorrelations (Boateng et al., 2018). Investigations of construct validity were also performed for SEIC to meaningfully overlap with self-efficacy constructs for listening and speaking skills typically used in classroom contexts (Hunsley and Meyer, 2003).

### Measures

#### Self-Efficacy in Intercultural Communication (SEIC; Peterson et al., 2011)

The full version of the SEIC is a 34-item self-reported measure that attempts to inductively summarize a domain of self-efficacy as it relates to intercultural communication. The original English version of the items was distributed to the assistant language teachers as native speakers of English, while the Japanese items were adapted, backtranslated into English, and distributed to the Japanese teachers of English and undergraduate students at two Japanese universities. The English and Japanese items used for the short form are available in Table 2, and the full form is available in Supplementary Material 1.

**TABLE 2:** Standardized loading estimates for the SEIC eight-item short form for a cross-cultural sample of adult teachers and undergraduate student participants.

Item	SEIC Short Form	Teachers (*n* = 861)		Undergraduate Students (*n* = 266)	
	English and Japanese	*Standardized Loading*	*Standardized Error*	*Standardized Loading*	*Standardized Error*
1	How well can you think possible outcomes through before you speak? 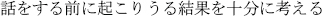	0.73	0.02	0.75	0.04
2	How well are you able to adapt to an interaction in which the topic changes from familiar to unfamiliar territory? 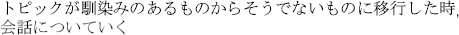	0.79	0.01	0.80	0.03
3	How well can you communicate with people who are in positions of authority? 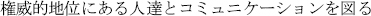	0.80	0.01	0.76	0.04
4	When in a face to face conversation, how well can you gauge what another person wants you to communicate? 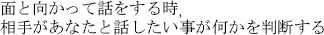	0.80	0.01	0.77	0.04
5	How well can you recognize subtle shades of meaning in an interaction? 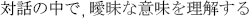	0.77	0.02	0.79	0.03
6	How well can you communicate in impromptu situations? 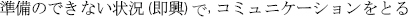	0.80	0.01	0.71	0.04
7	How well can you build consensus when you communicate? 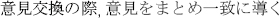	0.79	0.02	0.70	0.04
8	How well can you communicate with people you don’t like? 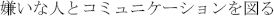	0.59	0.02	0.50	0.06

*SEIC, Self-Efficacy in Intercultural Communication.*

All items in the study by Peterson et al. (2011) began with “*How well can you*…,” and utilized a seven-point response scale semantically labeled at the poles, with 1 equating to “*not well at all*” and 7 equating to “*very well*” (personal communication with the authors, 2017). In this study, a six-point scale ranging from “*I definitely cannot do this*” to “*I can do this very well*” semantically labeling with all points along the scale was employed. This departure from the original study was made for three reasons. First, item response theory-based empirical evidence suggests deviation in psychological distance estimations between response categories increases as the number of response categories available increases, and that this deviation influences item values (Wakita et al., 2012). Second, the degree to which a neutral response option actually indicates a neutral response has been called into question (Kulas and Stachowski, 2009, 2013), and some Japan-based researchers explicitly advise against neutral response options and eliminate them from Likert-style instruments (Nemoto and Beglar, 2014). Third, the L2 Speaking Self-Efficacy and L2 Listening Self-Efficacy scales (described subsequently) each employ six-point response scales, without a neutral option, and with each point on the response scale explicitly labeled. As each of these instruments was delivered as part of a single survey battery, we determined that consistent use of a six-point response scale for the entire battery was both intuitive and methodologically sound.

#### L2 Speaking Self-Efficacy (S-SE; Hicks and McLean, 2014)

The S-SE is a 20-item instrument developed with Japanese university students. Technical item quality was evaluated with Rasch principal components analysis, and nomothetic span was investigated against the WTC model and constructs in its validation. In addition, external validity was previously investigated and supported for the items to discriminate from foreign language speaking anxiety. Items include can-do statements such as “I can respond in English to greetings from international students on campus.” Participants responded to a six-point scale ranging from “*I definitely cannot do it*” to “*I can definitely do it*.”

#### L2 Listening Self-Efficacy (L-SE; Kramer and Denison, 2016)

The L-SE is a 14-item L2 domain-specific instrument drawn from items by Burrows (2012) and fitted with Bandura (2006) prescriptions for self-efficacy scale development. The scale was validated for Japanese ESL learners achieving elements of content relevance from interview data, technical item quality from Rasch rating scale modeling, convergent validity through moderately positive correlations with vocabulary knowledge and divergent validity through moderately negative correlations with foreign language listening anxiety. Sample items include “If I heard an English conversation at the level of a junior high school textbook, I would understand it,” and “If I watched the news in English, I would understand it.” A six-point response scale ranging from “*I definitely cannot do it*” to “*I can definitely do it*” was used.

#### Intercultural Effectiveness (IES; Mendenhall et al., 2008)

The IES is a 60-item self-report measure developed to evaluate the overall competency of individuals when interacting with those from cultures that are different from their own. The IES was created as a simplified version of the Global Competencies Inventory (GCI), a line of research in which Mendenhall and Osland (2002) distilled the dozens of competencies hypothesized to influence global leadership effectiveness to a core set of six dimensions. Three of these dimensions (cross-cultural relationship skills, traits and values, and cognitive orientation) were found to overlap with the competencies critical to expatriate adjustment to living and working in a foreign country. The IES is simply a “less complex version of the GCI” (Mendenhall et al., 2008), comprised of three dimensions that each contain two subfactors. *Continuous Learning* (α = 0.85), which is operationalized as “the degree to which individuals engage the world by continually seeking to understand themselves and also learning about the activities, behavior, and events that occur in the intercultural environment” is comprised of the two subscales of Self-Awareness (α = 0.76) and Exploration (α = 0.82). *Interpersonal Engagement* (α = 0.85) is operationalized as “the ability to develop positive relationships with host-nationals” and is comprised of the two subscales *Global Mindset* (α = 0.84) and *Relationship Interest* (α = 0.80). Lastly, *Hardiness* (α = 0.84), operationalized as “people’s ability to effectively manage their emotions and stress, along with their ability to view other cultures and people from those cultures in positive ways and to be non-judgmental about ideas and behaviors that are new” is comprised of two subscales called Positive Regard (α = 0.79) and Emotional Resilience (α = 0.81). As the IES is a proprietary instrument, sample items are restricted from third-party reproduction. Participants responded to a five-point Likert scale that ranges from “*Strongly Disagree*” to “*Strongly Agree*.”

## Results

### Sample Characteristics and Reliability Analysis

The descriptive statistics and reliability of the study variables were calculated in JASP (JASP Team, 2018). Participants who fully completed the survey were retained, of which 266 participants were used for the examination of internal validity, while listwise deletion left 240 university students (53 males, 187 females) for analysis of external validity (University 1: *N* = 161, 67% female; University 2: *N* = 79, 100% female). According to conventional guidelines for reliability as estimated by Cronbach’s α and McDonald’s ω, values greater than 0.7 were favored and internal consistency was supported for the self-efficacy study variables (SEIC: α = 0.88; ω = 0.87; S-SE: α = 0.96; ω = 0.96; L-SE: α = 0.92; ω = 0.88).

### Confirmatory Factor Analysis

Confirmatory factor analysis was performed using the *lavaan* package (Rosseel, 2012) to evaluate structural validity. Listwise deletion to retain fully completed item-level response data left 861–876 teachers for the CFA. The default maximum likelihood estimator was used. Fit indices and information criteria were compared as model selection measures. The one-factor model with eight items as originally proposed as the short form from the sample of assistant language teachers as sojourners in Peterson et al. (2011) was fit for the present sample of teachers, and the original 34-item full form and eight-item short form were examined for the new targeted sample population of university students. Several indices of model fit were considered, namely, the chi-square (χ^2^), Comparative Fit Index (CFI), Tucker–Lewis Index (TLI), Goodness of Fit Index (GFI), Standardized Root Mean Square Residual (SRMR), and Root Mean Square Error of Approximation (RMSEA). Table 1.2 displays the fit indices from each procedure and sample configuration. Acceptable model fit was determined from a combined consideration of the incremental (CFI, TLI, GFI), absolute (SRMR), and parsimonious fit indices (RMSEA), such that CFI, TLI, and GFI values reached above 0.90 but especially exceeded 0.95, SRMR values were less than or close to 0.06, and RMSEA values were close to or less than 0.80 (Brown, 2014). Model comparison suggested that the one-factor model with eight items proposed by Peterson et al. (2011) provided acceptable fit for the ALTs (CFI = 0.973, TLI = 0.962, GFI = 0.943, SRMR = 0.028, RMSEA = 0.091), JTEs (CFI = 0.965, TLI = 0.951, GFI = 0.961, SRMR = 0.033, RMSEA = 0.082), and good fit for the teachers in total (CFI = 0.973, TLI = 0.963, GFI = 0.967, SRMR = 0.026, RMSEA = 0.078). While relatively higher RMSEA values were observed, model evaluation was considered in terms of overall goodness of fit. Extending to the desired sample of instrument use with university students, and in direct test of the proposed full-form and short-form structure by Peterson et al. (2011), the eight-item model (CFI = 0.919, TLI = 0.887, GFI = 0.901, SRMR = 0.050, RMSEA = 0.129) offered indications of better model fit over the 34-item model (CFI = 0.788, TLI = 0.775, GFI = 0.602, SRMR = 0.065, RMSEA = 0.107) in terms of model coverage and complexity, with the exception of RMSEA. The standardized factor loading estimates for the best fitting eight-item model are provided in Table 2. Loadings ranged from 0.50 to 0.80, suggesting factor determinacy among both samples of schoolteachers and university students. The full form of translated items and standardized loading estimates for the 34-item model are provided in Supplementary Material 1.

**TABLE 1.2:** Fit indices from confirmatory factor analysis of the SEIC in the samples of teachers and university students.

Model		*df*	Minimum Function Test Statistic (χ^2^)	χ^2^ *p*-value	CFI	TLI	GFI	SRMR	RMSEA (CI)
Sojourning Assistant Language Teachers (264)	8-item short-form model	20	63.867	0.000	0.973	0.962	0.943	0.028	0.091 (0.067–0.117)
Japanese Teachers of English (597)	8-item short-form model	20	100.813	0.000	0.965	0.951	0.961	0.033	0.082 (0.067–0.099)
Total Teachers (861)	8-item short-form model	20	129.452	0.000	0.973	0.963	0.967	0.026	0.078 (0.065–0.091)
Undergraduate Students (266)	34-item full model	527	1511.733	0.000	0.788	0.775	0.602	0.065	0.107 (0.100–0.113)
	8-item short-form model	20	74.791	0.000	0.919	0.887	0.901	0.050	0.129 (0.099–0.161)

*SEIC, Self-Efficacy in Intercultural Communication; CFI, Comparative Fit Index; TLI, Tucker-Lewis Index; GFI, Goodness of Fit Index; SRMR, Standardized Root Mean Square Residual; RMSEA, Root Mean Square Error of Approximation.*

### Measurement Invariance

Data from all samples were cross-validated through multigroup CFA-based MI procedures. For comparison of cross-cultural equivalence between the Japanese and English items, the *lavaan* package was applied to the data for ALTs and JTEs, with ALTs as the reference group. The results for model fit comparison can be seen in Table 3.1. Determination of the level of equivalence established was based on the smallest values for each information criterion. The values were lowest at the test of equal factor loadings [Akaike Information Criterion (AIC) = 17,896; Bayesian Information Criterion (BIC) = 18,901]. Further examination of model fit was conducted and depicted in Table 3.2. Comparative fit supported the level of loadings (CFI = 0.967; RMSEA = 0.080) over intercepts (CFI = 0.936; RMSEA = 0.105). Together, these results indicated support for adopting a level of metric invariance and comparable factor loadings between ALTs and JTEs and their respective language versions of the items.

**TABLE 3.1:** Fit indices for equivalence testing of the SEIC between ALTs and JTEs.

Measurement Invariance	*df*	AIC	BIC	χg^2^	χ^2^ difference	*df* difference	Pr (> χ^2^)
Configural	40	17,896	18,124	162.11			
Loadings	47	17,896	18,091	176.22	14.105	7	0.04935*
Intercepts	54	18,014	18,175	307.82	131.597	7	<2e-16***
Means	55	18,015	18,172	311.00	3.181	1	0.07450

Smaller values in information criteria support the level of measurement equivalence attained. Coefficients in bold *p < 0.05, ***p < 0.001. SEIC, Self-Efficacy in Intercultural Communication; ALT, assistant language teacher; JTE, Japanese Teacher of English; AIC, Akaike Information Criterion; BIC, Bayesian Information Criterion.

**TABLE 3.2:** Fit indices for equivalence testing of the SEIC between ALTs and JTEs.

Measurement Invariance	CFI	RMSEA	Δ CFI	Δ RMSEA
Configural	0.969	0.084		
Loadings	0.967	0.080	0.002	0.004
Intercepts	0.936	0.105	0.032	0.025
Means	0.935	0.104	0.001	0.001

*Smaller values in information criteria support the level of measurement equivalence attained. SEIC, Self-Efficacy in Intercultural Communication; ALT, assistant language teacher; JTE, Japanese Teacher of English; CFI, Comparative Fit Index; RMSEA, Root Mean Square Error of Approximation.*

To investigate the levels of equivalent measurement and performance of the Japanese items between JTEs as older adult teaching professionals and Japanese undergraduate students as younger emerging adult learners, another multigroup CFA-based MI procedure was conducted. The results for model fit comparison can be seen in Table 3.3. Again, determination of the level of equivalence established was based on the smallest values for each information criterion. The values were lowest at the test of equal factor loadings for the AIC (AIC = 17,784) but not the BIC (BIC = 179,179), which was lowest for the test of equal intercepts (BIC = 17,960). Thus, further model fit comparison was conducted and depicted in Table 3.4. A conservative interpretation of comparative fit suggested supporting the level of loadings (CFI = 0.948; RMSEA = 0.092) over intercepts (CFI = 0.941; RMSEA = 0.091). As the change in RMSEA was negligible, the change in CFI was used to arbitrate model selection, which favored support of equivalent loadings (ΔCFI = 0.001) over intercepts (ΔCFI = 0.036), in line with recommendations for ΔCFI as a goodness-of-fit index in MI (Cheung and Rensvold, 2002). Together, these results indicated support for adopting a level of metric invariance and comparable factor loadings for the eight indicators in Japanese between adult teaching professionals and emerging adult university student learners.

**TABLE 3.3:** Fit indices for equivalence testing of the SEIC between JTEs and university students who both responded to the items adapted in Japanese.

Measurement Invariance	*df*	AIC	BIC	χ^2^	χ^2^ difference	*df* difference	Pr (> χ^2^)
Configural	40	17,788	18,016	208.35			
Loadings	47	17,784	17,979	219.03	10.675	7	0.1534464
Intercepts	54	17,798	17,960	246.78	27.755	7	0.0002436***
Means	55	18,015	18,034	327.38	80.595	1	<2.2e-16***

Smaller values in information criteria support the level of measurement equivalence attained for the Japanese-adapted instrument. Coefficients in bold ***p < 0.001. SEIC, Self-Efficacy in Intercultural Communication; JTE, Japanese Teacher of English; AIC, Akaike Information Criterion; BIC, Bayesian Information Criterion.

**TABLE 3.4:** Fit indices for equivalence testing of the SEIC between JTEs and university students.

Measurement Invariance	CFI	RMSEA	Δ CFI	Δ RMSEA
Configural	0.949	0.099		
Loadings	0.948	0.092	0.001	0.007
Intercepts	0.941	0.091	0.036	0.001
Means	0.917	0.107	0.024	0.016

*Smaller values in information criteria support the level of measurement equivalence attained. SEIC, Self-Efficacy in Intercultural Communication; JTE, Japanese Teacher of English; CFI, Comparative Fit Index; RMSEA, Root Mean Square Error of Approximation.*

### Correlational Analysis

An initial correlational procedure was opted for examining the coverage of the short-form instrument in comparison to the original scale. As a result, the eight-item version strongly correlated with the full 34-item version (*r* = 0.94), suggesting that it could capture a majority of the variance in the parsimonious set of items proposed by Peterson et al. (2011). Next, Pearson’s correlation coefficients were examined for the study variables for the student dataset (*N* = 240). The results are depicted in Table 4. TOEIC scores as a measure of language proficiency did not correlate with the SEIC short form on factors of intercultural effectiveness (*r* = 0.08). Overall Intercultural Effectiveness moderately correlated in the positive direction for the short-form SEIC (*r* = 0.40), indicating valid overlap in the relevant domain of beliefs in intercultural competencies. Specifically, SEIC correlated with *Continuous Learning* (*r* = 0.36) and *Interpersonal Engagement* (*r* = 0.37) as component factors of the IES, but not with *Hardiness* (*r* = 0.06), suggesting boundary separation in the nomothetic span of the construct measured by the SEIC. Further examination of construct overlap was conducted. Correlations were compared for divergent and convergent relationships in a set of data from University 2 (*N* = 79) simultaneously allocated to measure L-SE, S-SE, and SEIC. The results are given in Table 5. Supported positive correlations were observed for L-SE (*r* = 0.42) and S-SE (*r* = 0.28), indicating that the listening self-efficacy skill domain demonstrated particular magnitude in the strength of the relationship for the sample.

**TABLE 4:** Pearson’s correlation coefficients for the proposed self-efficacy in intercultural communication short-form instrument and study variables for the university students (*n* = 240).

Measure	1	2	3	4	5	6
(1) Self-Efficacy in Intercultural Communication	—					
(2) TOEIC Total Score	0.08	—				
(3) *Continuous Learning*	0.36***	0.15*	—			
(4) *Interpersonal Engagement*	0.37***	0.16*	0.43***	—		
(5) Hardiness	0.06	0.08	0.15*	0.03	—	
(6) Overall Intercultural Effectiveness	0.40***	0.19*	0.78***	0.76***	0.49***	—
*M*	2.92	585	3.60	3.03	2.86	3.17
*SD*	0.82	147	0.42	0.46	0.36	0.29

Coefficients in bold *p < 0.05, ***p < 0.001.

**TABLE 5:** Pearson’s correlation coefficients for the proposed self-efficacy in intercultural communication short-form instrument and study variables for undergraduate students from University 2 (*n* = 79).

Measure	1	2	3	4
(1) Self-Efficacy in Intercultural Communication	—			
(2) TOEIC Total Scores	0.08	—		
(3) Speaking Self-Efficacy	0.28*	0.64***	—	
(4) Listening Self-Efficacy	0.42***	0.49***	0.75***	—
*M*	3.71	469	2.78	3.19
*SD*	0.84	173	0.79	0.81

Coefficients in bold *p < 0.05, ***p < 0.001.

## Discussion

This paper set out to address areas of validation for eight item indicators culled and explored by Peterson et al. (2011) by (1) securing internal validity considerations with careful confirmatory analysis of the eight-item short form in a study design that shares sample characteristics of culturally diverse sojourning language teachers and a new context of Japanese teaching professionals; and (2) attempting to localize and replicate good fit among psychometric properties and nomological networks in samples of undergraduate students from two universities in Japan that would serve as targeted populations for the use of the instrument. SEIC was examined with respect to relevant constructs for external validity.

### Internal Validity

As seen in the results for the standardized factor loadings suggesting factor determinacy, the eight items proposed as the short form by Peterson et al. (2011) yielded acceptable overall model fit (Tables 1.2–3.4). A notable caveat emerged in regard to RMSEA values, which exceeded recommended cutoff criteria for the university students. As the other indices were strongly within ranges that suggest good model fit, we surmise that this might be amenable to sample size limitations as our university student sample (*N* = 264) was relatively smaller, and higher RMSEA values can occur in spite of other strong indicators of overall model fit due to relatively smaller sample sizes (Brown, 2014). Moreover, the level of metric invariance suggested by the smallest fit indices also shows that the items perform comparably between sojourning teachers and Japanese schoolteachers, as well as between adult teaching professionals and emerging adult student learners. As acceptable fit was observed for the versions administered in English for ALTs and in Japanese for JTEs, the results offer a degree of multilingual forms support for the items as well as a degree of cross-cultural validity for the factor structure between groups (Aresi et al., 2018). This observation of structural validity extended from teachers to students as well, supporting the form of the instrument adapted for Japanese students. Furthermore, the eight-item version highly correlated with the 34-item full version administered to students, indicating that most of the variance was recoverable in a smaller subset of items as proposed by Peterson et al. (2011). For students whose model fit was acceptable for many fit indices, but marginal in regard to RMSEA, the full list of Japanese items has been appended (Supplementary Material 1) for future researchers to examine characteristics of the factor structure in larger samples of target groups (i.e., to find better parsimony among university student samples). Overall, the factor structure was specified and confirmed by these findings in new data of teaching professionals in intercultural contexts as proposed in the original validation and even supported with degrees of observable measurement equivalence, which suggests that the eight-item instrument possesses internal validity.

### External Validity

For external validity, the SEIC as a latent variable demonstrated moderately positive intercorrelations with two out of the three primary factors of intercultural effectiveness (*Continuous Learning* and *Interpersonal Engagement*) measured at pretest to a study abroad tour, but not with self-reported TOEIC as a measure of language proficiency (Table 4). Correlations with Overall IES as a standard measure also showed that elements of this domain can be captured parsimoniously with these eight independently derived items. This indicates that the SEIC construct measured connects to theoretically justified content domains in the nomological net of constructs related to beliefs of intercultural effectiveness. Furthermore, in a check of concurrent validity from the sample of university students from an all-female university, the construct moderately correlated with the L-SE as a measure of beliefs about receptive skills and S-SE as a measure of beliefs about productive skills (Table 5). These findings suggest that the SEIC as a construct measurably overlaps with self-efficacy competencies for language skill domains. The following sections discuss the implications of these findings for self-efficacy in intercultural communication for applied disciplines.

### Self-Efficacy in Intercultural Communication as Competency in Positive and Educational Psychology Settings

As positive traits and abilities form one of the pillars of positive psychology (Seligman, 2004), SEIC showed some evidence of spanning relevant content domains as a competency belief. The correlational findings for the IES (Table 4), L-SE, and S-SE (Table 5) thus provided insights for theorizing and modeling efforts and applications to positive education, simulations, and intercultural communication programming contexts.

Overall IES positively correlated with SEIC with a moderate magnitude indicating nomothetic span and provided indications of factor specificity with implications for applied positive psychology constructs. *Continuous Learning* consists of the subfactors self-awareness and exploration. Respondents with higher self-awareness typically possess an acute understanding of that which they can and cannot do, and this understanding informs their capacity to continuously, and strategically, acquire new skills. In this way, the content of *Continuous Learning* overlaps with task related self-efficacy as control over learning, and provides a straightforward interpretation for the content of the SEIC. The relationship between the SEIC and *Continuous Learning* suggests that the construct might suitably be applied to self-regulated learning contexts for intercultural communication skills. The correlation between student SEIC and *Interpersonal Engagement* is suggestive as the underlying dimension in the latter consists of two subfactors: *Global Mindset* and *Relationship Interest*. Those with higher *Global Mindset* scores are essentially those who have a stronger interest in actively expanding their knowledge of other cultures, as well as a sense of cosmopolitanism that facilitates their adjustment to a foreign culture. *Relationship Interest* involves actively choosing to build meaningful relationships with people from cultures outside our own. *Interpersonal Engagement* as a measure of positive relationships and interactions with host nationals suggests that the “R” positive psychology domain in the PERMA profiler and its framework extensions and configurations might be a source of positive construct representation.

In contrast, the IES factor of *Hardiness*, which contains straightforward positive psychology elements such as positive self-regard and emotional resilience, did not show a strong supported relationship to SEIC and shows an almost discriminant and lowly supported pattern away from content domains that would typically be represented by self-concepts in applied positive psychology. This is a surprising inferential distinction given the self-oriented evaluative nature of self-efficacy and suggests that SEIC is limited in its scope as a positive learner belief. However, the IES approach to measuring *Hardiness* might differ from the measurement philosophy of constructs such as grit, which leave the door open to investigating SEIC with conventionally operationalized positive traits.

The lack of association might also suggest that the domain captured for pre-study abroad Japanese students is specified not at the level of *Hardiness* as a dispositional characteristic but at the level of component communication skills, as evinced by relationships to L-SE and S-SE in Table 5. The observed delineation for relative contributions of the L-SE and S-SE self-efficacy domains is particularly salient considering Fantini’s (2009) argument that intercultural communicative competence “may be defined as complex abilities that are required to perform effectively and appropriately when interacting with others who are linguistically and culturally different from oneself… whereas effective reflects the view of one’s own performance in the target language-culture… [and] appropriately reflects how natives perceive such performance” (p. 458). Thus, intercultural communicative competence is typified by a dynamic set of skills, rather than the skills in isolation. Language and culture are frequently inseparable in cross-linguistic and cross-cultural interactions, where effectiveness is determined by the self and appropriateness is determined by others. In this manner, this relational dynamic supports the notion that the “R” component of the PERMA model for interpersonal relationships in applied positive psychology is a likely candidate for counterpart considerations in applied linguistics extending to the crossover construct of the SEIC and might play a role in the reason for the low strength in relationship to measured levels of *Hardiness* relative to other factors operationalized by the IES. However, the factor itself may not show differences in study abroad exposure. Notably, Nguyen (2017) did not observe changes in *Hardiness* from a short-term study abroad pre–post design, suggesting that the competency factor may not be sensitive to change in these contexts or could depend on factors that rely on a depth of sociocultural adjustment.

Students who have been studying English with some sense of purpose and an eventual goal of studying abroad are represented in our samples. It may be possible that students are motivated to use learned languages effectively and study abroad to compare their experiences in self-concept with those of the outside world as a form of adolescent development or sociocultural identity affirmation or formation. Classroom experiences meeting real-world experiences then serve to connect and reinforce these outcomes especially related to linguistics skill competence, whose outcomes harmonize with those described as desired by policy-making institutions as a “fundamental competency for working people” (Yonezawa, 2014). We argue that SEIC is a construct that crosses the borders between the disciplines, and the presented findings suggest coverage of the SEIC for interpersonal outcomes as a noted advantage among such samples as the likely population for instrument use. Intercultural training through simulations and positive education designs for lessons or programs in cultural competence might benefit from examinations of SEIC, and the construct might extend to signature strengths such as transcendence and aesthetic appreciation (Seligman, 2004) in the event of traveling abroad. It seems plausible that transformative experiences from exposure to the expansions in worldliness associated with sojourns could meaningfully relate to the domain of SEIC and spiral upward in relationships to desired global competencies.

Overall, at present, our findings indicate some potential for the SEIC to identify individuals who might possess higher baseline IES dimensions of *Continuous Learning* and *Interpersonal Engagement*, which are hypothesized to map on to traits such as openness to, respect for, and curiosity about other cultures, and are widely believed to be conducive to developing intercultural competence (Deardorff, 2006). Future research perhaps using goal-related theories like self-determination theory (Lee and Bong, 2019; McEown and Oga-Baldwin, 2019) more conventionally measured positive psychology constructs (Dewaele et al., 2019) plausibly extending to authentic personality for students (Wood et al., 2008) or PERMA in the workplace (Watanabe et al., 2018) for professionals might be useful directions to further investigate these relationships.

### Self-Efficacy in Intercultural Communication as Positive Trait in Applied Linguistics

The four skills of reading, writing, listening, and speaking encode and decode language in ways that convey intelligible meaning to interlocutors under the duress of the specious present. For receptive skills, verbalized strategies for listening from social modeling have been put forth as a mechanism for augmenting self-efficacy (Schunk and Rice, 1984). Listening-related self-efficacy was also proposed to boost confidence in parsing and responding to authentic oral input for students learning English for academic purposes, especially in lessons that incorporated feedback and interpersonal skills with reflective or dialogic approaches (Graham, 2011). The results in Table 5 for correlations with S-SE and L-SE suggested that the construct measured by the SEIC could extend its incremental validity through established relationships to classroom-relevant self-efficacy content domains, which crucially contain granular opportunities to build linguistic skill competence and experience with intercultural communication.

The relationship between SEIC and the S-SE points to transactional acts of communication as the items involve the respondent’s capacity to produce language given a context in which there is a goal, but the interlocutors are static in terms of attitudinal and sociolinguistic features. The items included on the L-SE instrument frame the respondent as a passive consumer of information they hear, rather than as part of an active conversation. As mentioned, the SEIC correlations with L-SE with S-SE are an important indicator of skill overlap. However, while the S-SE and L-SE were found to correlate convergently, we did not observe a significant correlation between SEIC and self-reported TOEIC. This reveals the possibility that student respondents may not conflate their TOEIC score or their domain-specific, language-skills-in-a-vacuum sense of speaking or listening self-efficacy, with the intermediate order level of real-world SEIC that the SEIC scale measures. An explanatory model of moderating factors with structured equations or regression analysis might be especially insightful to test the directions of these relationships and is planned for future research.

## Limitations and Future Directions

Real-world interaction is dynamic, requiring both listening and speaking skills to be utilized simultaneously, frequently under temporal, emotive, and sociocultural pressures, with consequences for the relative success or failure of the interactive sequence. Thus, as intercultural communication, speaking and listening do not occur in a vacuum, an observation of changes in SEIC over time remains the most pressing need for future research on the construct (Hammer, 2012; Vande Berg et al., 2012; Varela, 2017). Whether or not the construct is sensitive to post-sojourn or post-positive intercultural education program change is the major limitation in this report (Green and Olson, 2008; Deardorff and Arasaratnam-Smith, 2017; Varela, 2017). Additionally, while the correlations to relevant constructs such as L-SE and S-SE especially suggested theoretical overlap, the present study focused on especially pre-study abroad-bound students and differences in the relationships or dynamics of the SEIC construct might emerge in explicitly classroom-centered contexts. Further investigations of predictive validity remain necessary to determine whether individuals can undergo meaningful changes in their SEIC from learning experiences such as travel featuring high doses of L2 interactive behavior or upon engaging in classroom activities that place a heavy emphasis on intercultural interactions. Future studies might implement cross-lagged study designs that control for gradations in exposure to programs like study abroad, intercultural simulations, or collaborative online international learning and track changes in SEIC. For example, these lines of inquiry might situate the construct as useful to test the *investment* component of the EMPATHICS model by Oxford (2016), such that modeling could find that sojourners with higher (or lower) SEIC scores choose to engage more (or less) while abroad in a dose-responsive manner. Additionally, future studies could use the SEIC short form to determine whether aspects of personality as independent variables (Dewaele, 2012) are responsible for fostering greater or lesser engagement with host-nationals or interacting partners in intercultural learning programs. Investigators of the role of SEIC as a factor in professional self-efficacy and positive psychology might also meaningfully apply the tool with PERMA profiler and framework for the workplace (Watanabe et al., 2018). In sum, while more validation remains to be done, the present study offers degrees of internal and external validity for the domain of interest to SEIC.

## Conclusion

This study validated many open questions on the properties of the SEIC and offers a tool with valid indicators for researchers and practitioners who aim to observe self-efficacy in positive education and international programs that intersect with the domain of language learning and intercultural communication. The instrument is fit and the construct is poised for the domain as L2 intercultural communication has many opportunities for experiencing enhancement and mastery in linguistic skill competence as a positive learner competency. The observed relationships for SEIC suggest that task-related speaking and listening activities could make it outside of the classroom and into in-the-wild instances of intercultural communication that might occur in transformative learning experiences such as study abroad. We offer our localized adaptation of the tool as a valid instrument for further research and assessment purposes with these intentions, especially in Japan. For research purposes, this would make the instrument and construct a plausible candidate for experimental manipulation in programming with learning experiences that center around opportunities to develop positive traits especially tailored for university students.

## Data Availability Statement

The raw data supporting the conclusions of this article will be made available by the authors, without undue reservation.

## Ethics Statement

Ethical review and approval was not required for the study on human participants in accordance with the local legislation and institutional requirements. The patients/participants provided their written informed consent to participate in this study.

## Author Contributions

RK served as the primary author of the manuscript, contributed to conceptualization, methodology, software, formal analysis, validation, original draft preparation, data curation, and review and editing. AS provided funding acquisition, project administration, investigation and data collection, formal analysis, and revisions. Both authors contributed to the article and approved the submitted version.

## Conflict of Interest

The authors declare that the research was conducted in the absence of any commercial or financial relationships that could be construed as a potential conflict of interest.
