# Supplementary material for: Interacting With Competence: A Validation Study of the Self-Efficacy in Intercultural Communication Scale-Short Form
Source: Front Psychol. 2020 Sep 4;11:2086. doi: 10.3389/fpsyg.2020.02086 (PMC7498699; doi:10.3389/fpsyg.2020.02086)
Supplement: Supplementary file 1 [file Table_1.DOCX]

**Supplementary Material 1.** *Item indicators and standardized loading estimates for the full-form 34-item SEIC used with Japanese university student participants. English items adopted from Peterson et al. (2011) and translated Japanese items are provided below.*

| Item | Self-Efficacy in Intercultural Communication  英語を話す環境で、あなたは以下の事柄をどのくらい上手にできますか。 | Undergraduate Students  (*n* = 266) |  |
| --- | --- | --- | --- |
|  |  | *Standardized Estimate* | *Standardized Error* |
| 1 | How well can you introduce new or different ways of solving a problem in an interaction? 対話の中で問題解決の新しい、または違った方法を提示する | 0.736 | 0.037 |
| 2 | How well can you ask questions to get what you need? 必要なものを得るために質問する | 0.663 | 0.045 |
| 3 | How well can you infer or guess at the meaning of messages in an interaction? 対話の中でメッセージの意味を推測したり察したりする | 0.699 | 0.041 |
| 4 | How well can you inspire others to gain new insight when you communicate with them? コミュニケーションを図る中で、他者に新しい洞察力を身につけたいと思わせる | 0.694 | 0.041 |
| 5 | How well can you invent new words or phrases to illustrate unique circumstances? ユニークな状況を説明するために、新しい言葉やフレーズを考え出す | 0.617 | 0.049 |
| 6 | How well can you stand up in a group of people and give your opinion? 集団の中で自分の意見を発言する | 0.746 | 0.036 |
| 7 | How well can you think possible outcomes through before you speak?  話をする前に起こりうる結果を十分に考える | 0.715 | 0.039 |
| 8 | How well are you able to adapt to an interaction in which the topic changes from familiar to unfamiliar territory? トピックが馴染みのあるものからそうでないものに移行した時、会話についていく | 0.737 | 0.037 |
| 9 | How well can you communicate with people who do not share your language? 母国語が違う人達とコミュニケーションを図る | 0.793 | 0.030 |
| 10 | How well can you explain abstract concepts? 抽象的な事柄を説明する | 0.722 | 0.038 |
| 11 | How well can you communicate with people who are in positions of authority? 権威的地位にある人達とコミュニケーションを図る | 0.723 | 0.038 |
| 12 | How well can you assert your opinion when you communicate? 話をする時に自分の意見をはっきり言う | 0.755 | 0.035 |
| 13 | How well can you communicate when people are trying to intimidate you? あなたを威圧しようとする人々とコミュニケーションを取る | 0.703 | 0.040 |
| 14 | When in a face to face conversation, how well can you gauge what another person wants you to communicate? 面と向かって話をする時、相手があなたと話したい事が何かを判断する | 0.768 | 0.033 |
| 15 | How well can you recognize subtle shades of meaning in an interaction? 対話の中で、曖昧な意味を理解する | 0.714 | 0.039 |
| 16 | How well can you communicate in a discussion criticizing your own culture? 自分の文化を批判されているような議論の中で、コミュニケーションを図る | 0.762 | 0.034 |
| 17 | How well can you communicate with people from different cultures within your own country? 自分の国にいる異文化出身の人々とコミュニケーションをとる | 0.737 | 0.037 |
| 18 | How well can you gauge the appropriate manner in which you are expected to communicate in an interaction (for instance, in a job interview)? 対話（例えば就職の面接など）であなたに求められる適切な話し方を判断する | 0.696 | 0.041 |
| 19 | How well can you shift between words and body language to get your point across? 言いたい事を円滑に伝えるために、言葉にしたり身振りを使ったりと、伝達手段を変える | 0.720 | 0.039 |
| 20 | How well can you predict what another person will say in an interaction? 会話の中で相手の言いたい事を予測する | 0.803 | 0.029 |
| 21 | How well can you listen when you are in a communication situation? 意見交換が必要な状況で、相手の話を聞く | 0.749 | 0.035 |
| 22 | How well can you communicate in impromptu situations? 準備のできない状況（即興）で、コミュニケーションをとる | 0.786 | 0.031 |
| 23 | How well can you communicate with people from different countries? 出身国の違う人々とコミュニケーションを図る | 0.804 | 0.029 |
| 24 | How well can you recognize whether it is appropriate to engage in confrontation? 意見が衝突した時、議論することが適切か理解する | 0.789 | 0.030 |
| 25 | How well can you see things from another person’s point of view in an interaction? 会話において、別の人の視点から物事を見る | 0.744 | 0.036 |
| 26 | How well can you take criticism? 批判を受け入れる | 0.635 | 0.048 |
| 27 | How well can you communicate your agreement or disagreement in an argument? 議論の際、賛成・反対意見を伝える | 0.771 | 0.033 |
| 28 | How well can you communicate with people with disabilities?障害のある人々とコミュニケーションを図る | 0.671 | 0.044 |
| 29 | How well can you communicate with people who are of a significantly different age than you? かなりの年齢差のある人とコミュニケーションを図る | 0.687 | 0.042 |
| 30 | How well can you use humor in your communication? コミュニケーションの中でユーモアを交える | 0.687 | 0.042 |
| 31 | How quickly can you find common ground with others when you communicate? 会話をするとき、素早く他者との共通点を見つける | 0.796 | 0.030 |
| 32 | How well can you communicate with people you strongly disagree with? 全く意見の違う人々とコミュニケーションを取る | 0.817 | 0.027 |
| 33 | How well can you build consensus when you communicate? 意見交換の際、意見をまとめ一致に導く | 0.702 | 0.041 |
| 34 | How well can you communicate with people you don’t like? 嫌いな人とコミュニケーションを図る | 0.605 | 0.050 |
